# An evaluation of the impact of ‘Lifeskills’ training on road safety, substance use and hospital attendance in adolescence

**DOI:** 10.1016/j.aap.2015.10.017

**Published:** 2016-01

**Authors:** Alison Teyhan, Rosie Cornish, John Macleod, Andy Boyd, Rita Doerner, Mary Sissons Joshi

**Affiliations:** a School of Social and Community Medicine, University of Bristol, UK; b Department of Psychology, Social Work & Public Health, Oxford Brookes University, UK

**Keywords:** Safety training, Accident prevention, Pedestrian skills, Cycle safety, Adolescence, ALSPAC

## Abstract

**Purpose:**

To evaluate if attendance at Lifeskills, a safety education centre for children in Year 6 (10–11 years), is associated with engagement in safer behaviours, and with fewer accidents and injuries, in adolescence.

**Methods:**

The sample are participants in the Avon Longitudinal Study of Parents and Children who attended school in the Lifeskills catchment area in Year 6; 60% attended Lifeskills. At 14–15 years, participants (n approximately 3000, varies by outcome) self-reported road safety behaviours and accidents, and perceived health effects and use of alcohol, cannabis, and tobacco. Additional outcomes from linkage to Hospital Episodes Statistics were available for a sub-sample (n = 1768): hospital admittance (for accident-related reason, from 11–16 years) and A&E attendance (for any reason, from approximately 14–16 years).

**Results:**

Children who attended Lifeskills were more likely to report using pedestrian crossings on their way to school than children who did not attend (59% versus 52%). Lifeskills attendance was unrelated to the ownership of cycle helmets, or the use of cycle helmets, seat belts, or reflective/fluorescent clothing, or to A&E attendance. Use of cycle helmets (37%) and reflective/fluorescent clothing (<4%) on last cycle was low irrespective of Lifeskills attendance. Lifeskills attendance was associated with less reported smoking and cannabis use, but was generally unrelated to perceptions of the health impact of substance use.

**Conclusions:**

Lifeskills attendance was associated with some safer behaviours in adolescence. The overall low use of cycle helmets and reflective/fluorescent clothing evidences the need for powerful promotion of some safer behaviours at Lifeskills and at follow-up in schools.

## Highlights

• Lifeskills training is for 10–11 year olds and covers many safety domains.
• Evaluated using longitudinal cohort data linked to hospital attendance data.
• Attendance at A&E common but hospital admissions coded as due to an accident rare.
• Lifeskills attendance associated with some reported safer behaviours in adolescence.
• Use of cycle helmets and reflective/fluorescent clothing low overall.

## 1 Introduction

Adolescence is a developmental period during which many risk-taking behaviours emerge, increase and eventually peak (Boyer, 2006). The term risk-taking generally refers to behaviours associated with some probability of undesirable results, and can include poor road safety, substance use, and unsafe sexual activity (Boyer, 2006, Beyth-Marom and Fischhoff, 1997). Risky behaviours can be actions (e.g. drinking alcohol) or non-actions (e.g. not wearing a cycle helmet) (Beyth-Marom and Fischhoff, 1997). Unintentional injuries during adolescence are a major cause of morbidity, and the leading cause of mortality, both in England (Fauth and Ellis, 2010) and worldwide (Peden, 2008). Since unintentional injury is not only costly to individuals and their families but also to the state, prevention is a public health priority (Lyons et al., 2011, Public Health England, 2014, Royal Society for the Prevention of Accidents, 2013). Education has long been regarded as important in the prevention of injuries and substance use in children and young people. However, whilst there is some evidence that education increases safety knowledge, evidence of whether there are subsequent reductions in injuries or accidents is more limited (Fauth and Ellis, 2010).

The Lifeskills safety education centre in Bristol opened in 2000 and is one of seventeen permanent LASER (Learning About Safety by Experiencing Risk) projects in the UK (Lifeskills, 2015). It is built as a realistic village comprising a number of ‘sets’ including a road, houses, river, and railway line (Table A.1 summarises learning objectives by set). Typically, children visit the centre in whole class groups and during each school's 2-h visit, trained adult Volunteer Guides take pupils around the sets of the village in small groups of 3–4 pupils. The children work through interactive, safety-related activities with their Guide, and are given the opportunity for discussion. This interactive and experiential approach is viewed as good practice (Mcwhirter and Francis, 2012, Tolmie et al., 2005).

To achieve accident avoidance or engage in safe behaviours, risk needs to be recognised and knowledge of appropriate behaviours needs to be acquired, retained and put into action. Safety education interventions need to be evaluated from all these points of view (House of Lords Science and Technology Select Committee, 2011). An early evaluation of Lifeskills assessed knowledge pre-intervention and at three time points post-intervention to distinguish between immediate learning and longer-term retention. Good acquisition and retention was shown in many but not all domains (Lamb et al., 2006, Cowburn et al., 2003). However, the early evaluation did not investigate whether Lifeskills training was associated with engagement in safer behaviours, or a reduction in accidents and injury, over time.

The current research uses data from ALSPAC, a longitudinal study based in the same geographic area as Lifeskills, to evaluate the long-term effectiveness of some aspects of the Lifeskills training by comparing outcomes for children who did and did not attend. This evaluation focuses on outcome measures where achievement of the Lifeskills learning objectives would be expected to influence the outcome. These measures were chosen a priori by the study team and related to road safety, perceptions and use of substances (alcohol, tobacco, cannabis), and hospital attendance.

## 2 Methods

### 2.1 Sample

Subjects were participants in the Avon Longitudinal Study of Parents and Children (ALSPAC). Details of the ALSPAC study have been published (Boyd et al., 2013) and a searchable data dictionary is available (www.bristol.ac.uk/alspac/). ALSPAC recruited pregnant women with expected delivery dates between April 1, 1991 and December 31, 1992 who lived in a defined geographic area (Avon, UK). There were 14,062 live births and 13,988 children alive at one year. The children have been studied throughout their lives using maternal and self-report questionnaires, and clinic visits. Singleton children who were registered at a state-maintained school in the Lifeskills catchment area in Year 6 (as recorded in the National Pupil Database [NPD]) were eligible for inclusion in the current study (*n* = 10,112) (Fig. A.1). Ethical approval for ALSPAC was obtained from the ALSPAC Ethics and Law Committee and the Local Research Ethics Committees.

### 2.2 Measures

#### 2.2.1 Exposure – lifeskills attendance

Schools in the Lifeskills catchment area (counties of Bath and North East Somerset, Bristol City, North Somerset, and South Gloucestershire) are eligible to book a visit for their Year 6 pupils, and each year there is the capacity for around 65% of schools to attend. Lifeskills provided an attendance register of schools that had visited during the academic years that the ALSPAC participants were in Year 6 (2001/2002, 2002/2003, and 2003/2004). The NPD Year 6 school registration details of the ALSPAC participants were linked to the Lifeskills school attendance register. Where a link was established, the child was classified as having attended Lifeskills; where no link was established, the child was classified as not having attended Lifeskills.

#### 2.2.2 Outcomes

##### 2.2.2.1 Road safety

The road safety related items were included in postal questionnaires sent to participants at age 14 years: own a cycle helmet (no, yes); wore a cycle helmet last time you rode a bike (no/can’t remember, yes); wore reflective/fluorescent clothing last time you rode a bike (no/can’t remember, yes); use of pedestrian crossings on the way to school (always/most times if available, sometimes/hardly ever/never); used a seat belt last time you travelled in a car (no/can’t remember, yes); always wear a seatbelt when in car (no, yes); involvement in a road traffic accident (RTA) in the past year as a pedestrian or cyclist (no, yes).

##### 2.2.2.2 Substance perceptions and use

Perception of the impact of alcohol [regular (daily) and binge drinking], regular smoking of cigarettes, and regular cannabis use, on physical and mental health were reported via a postal questionnaire sent at age 14 years. Answer options were on a 5-point scale from ‘very harmful’ to ‘very helpful’. A binary outcome was derived for each outcome (very harmful or not) as the vast majority reported each substance was harmful or very harmful.

Substance use was reported at age 15.5 years during a computer session administered at the ALSPAC clinic. The binary (no/yes) substance use variables were: frequent drinking (20 or more occasions in past 6 months); regular binge drinking (5+ drinks in any 24-h period in previous 2 years on 20+ occasions); behavioural problems due to alcohol (any of: used alcohol in dangerous situations; been accidentally physically hurt whilst drinking; had problem with police; got into fights because of drinking); recent smoking (any in past 30 days); current weekly smoking; occasional cannabis use (used more than once); problematic cannabis use (reported fairly often/very often to one or more of the 6 questions from the Cannabis Abuse Screening Test (Piontek et al., 2008)).

##### 2.2.2.3 Hospital attendance

A sub-sample of the ALSPAC cohort has been linked to the Hospital Episodes Statistics (HES) dataset compiled by the NHS Health and Social Care Information Centre (© 2012, re-used with the permission of The Health and Social Care Information Centre, all rights reserved.) This sub-sample is restricted to ALSPAC participants who, via a postal consent campaign conducted from 2011 to 2013, explicitly consented to the extraction and use of their NHS health records by ALSPAC. Of the 10,112 children who attended school in the Lifeskills catchment area in Year 6, 9284 were sent a consent pack requesting permission to link to their health records. Of these 24.6% (2282/9284) provided consent by December 18, 2012 (the last date on which HES records were extracted by ALSPAC). HES records were identified and extracted for 99.2% (2263/2282) of this sub-sample, of which 1762 had complete confounder data. The HES extracts include records of all hospital admissions and all A&E attendance episodes. For the hospital admittance outcome, admissions for reasons relevant to Lifeskills were identified using ICD-10 codes (V1-V49 road traffic accident related; F10-F19 alcohol, tobacco, drug related; T20-T30 burns and corrosions; W61-W74 drowning and submersions; T54.4, W85/W86 electrical accident) (World Health Organization, 2010). The binary outcome (no, yes) was hospital admittance for any of these reasons from the August of the year the child finished Year 6 until the August of the year they finished Year 11 (approximately age 11–16 years).

For A&E visits, the binary outcome (no, yes) was any A&E attendance from April 2007 (data only available from this date) until the August of the year the child completed Year 11. Therefore, for the oldest year group, the A&E data covers a 5 month period from April to August 2007; for the middle year group, a 17 month period from April 2007 to August 2008; and for the youngest year group, 29 months from April 2007 to August 2009.

#### 2.2.3 Potential confounders

Measures of socioeconomic position (SEP) were reported by the mother during pregnancy: highest maternal education (university degree; A level; O level; vocational/none), and highest parental occupational social class (higher of mother and her partner) based on the job codes of the Office for Population Censuses and Surveys (Office of Population Censuses & Surveys, 1991) and grouped into 4 categories (I/II [professional/managerial & technical]; IIInm [skilled, non-manual]; IIIm [skilled, manual]; IV/V [semi-skilled/unskilled manual]). Child variables included sex and age at data-collection. Whether or not the child had ever received cycle proficiency training (no, yes) was reported by the child at age 14 years and included as a potential confounder in analyses of the cycle-related outcomes. For A&E attendance, analyses were also adjusted for academic year because the time period included increased for each academic year. Two cluster variables were derived: school attended in Year 9 (age 14 years, i.e. close to the age of outcome data collection) and neighbourhood (Lower Super Output Area) of residence at the time of completion of the questionnaires at 14 years (Office for National Statistics).

### 2.3 Analyses

For each outcome, a separate complete case analysis was performed i.e. for each outcome, only children with data for that outcome, plus confounder data were included (see Table A.2 for a comparison of the study sample and those excluded due to missing data). Analysis of cycle helmet ownership was restricted to children who owned a bike, and analysis of cycle helmet use to those who owned a bike and helmet. Analysis of use of pedestrian crossings was restricted to participants who crossed at least one road on the way to school (Fig. A.1).

Multilevel logistic regression was used. Models were two level (individual at level 1, school at level 2) for most outcomes. A cross-classification of school and neighbourhood at level 2 was used for cycle helmet ownership, and use of helmets, pedestrian crossings, and seat belts, as there was statistical clustering at both the neighbourhood and school level for these outcomes. Interactions between Lifeskills attendance and sex, and Lifeskills attendance and maternal education and occupational social class, were tested for each of the outcomes.

## 3 Results

Approximately 60% of the children in the study sample had attended Lifeskills. Children who attended were more likely to be female, less likely to have a degree educated mother, and more likely to have had cycle proficiency training than those who did not attend (Table 1). Associations between Lifeskills attendance and each of the outcomes did not differ by sex, maternal education, or occupational social class and so models are presented adjusted for, rather than stratified by, sex and SEP. Descriptive results for each outcome are presented for the whole study sample. Further details on gender and SEP differences in the reporting of outcomes are available online (Tables A.3–A.5).

Cycle helmet ownership was reported by over 60% of adolescents who owned a bike. However, less than 40% of those who owned a bike and helmet reported that they had worn a helmet on their last cycle. Few bike owners had worn reflective or fluorescent clothing on their last cycle ride (<4%). Over half (56%) of the adolescents reported that they always or almost always used pedestrian crossings when crossing roads on their way to school. Seat belt use when last in a car was high, although 10% reported that they did not always wear one. Involvement in an RTA as a pedestrian or cyclist in the previous 12 months was rare (1.5%). Of these road safety related outcomes, Lifeskills attendance was only associated with the use of pedestrian crossings (Table 2). This association remained after adjustment for age, sex and SEP, and taking into account clustering at the neighbourhood and school level. However, the absolute difference between the groups was small (59% versus 52%).

The majority of adolescents perceived smoking and regular drinking in particular to be very harmful to physical health, and cannabis to be very harmful to both mental and physical health. Those who attended Lifeskills were more likely to report that binge drinking had a very harmful effect on health than those who did not attend Lifeskills (Table 3). This difference remained in adjusted analyses. For regular drinking, smoking and cannabis use, the proportion who reported that it was very harmful to either physical or mental health was consistently higher in those who attended Lifeskills, but absolute differences were small.

A proportion of the adolescents reported alcohol and tobacco use at age 15.5 years: 18% were frequent drinkers and 15% were recent smokers (had smoked at least once in past 30 days). The percentage who reported occasional cannabis use was lower (9%). Those who attended Lifeskills were less likely to be recent smokers than those who did not attend Lifeskills, were less likely to have smoked in the past week, and were less likely to be occasional cannabis users. These differences remained after adjustment for sex, age, and SEP (Table 4).

Only 15 of the participants with linked hospital records data had been admitted to hospital for one of the included reasons; the majority of the 15 had attended Lifeskills. Due to small numbers, it is not possible to present more details on these data. A&E attendance (for any reason) was relatively common, and did not differ by Lifeskills attendance status: 20.7% (226/1091) of those who had attended Lifeskills and 22.0% (149/677) of those who had not (adjusted OR 0.84, 95% CI 0.65 to 1.08).

## 4 Discussion

There is some evidence that children who attended Lifeskills at the age of 10–11 years had enhanced safety skills and attitudes in adolescence. Attenders were more likely to report that they always/mostly used pedestrian crossings on their way to school, were more likely to report that binge drinking was very harmful to physical health, and were less likely to report recent smoking or occasional cannabis use. However, for many of the outcomes, we did not detect differences between those who had and had not attended Lifeskills.

Of the road safety related outcomes, the use of pedestrian crossings was the only one to be associated with Lifeskills attendance. Choosing where to cross the road is something most of the children had some autonomy over (the majority walked with friends, siblings, or on their own; few walked with adults). Improving road safety on the school commute is a key priority as most road accidents involving children occur during school commuting times: an average of 16 children per week were killed or seriously injured between 8–9 am and 4–7 pm in England between 2008 and 2012, with those from more deprived neighbourhoods being most at risk (Public Health England, 2014). Lifeskills training aims to influence a child's road crossing behaviour through teaching children to recognise the risk posed by cars, that cars have long stopping distances, and that pedestrian crossings are the safest place to cross. These safety messages are pertinent to the school commute as research suggests that car drivers frequently do not comply with speed limits in school zones (Strawderman et al., 2015, Ellison et al., 2011). In the earlier evaluation of Lifeskills, knowledge of car stopping distances was one of the best acquired and retained pieces of information (Lamb et al., 2006).

Children aged 10–15 years are at greater risk of having a cycling accident than any age group other than adults aged over 60 years (Royal Society for the Prevention of Accidents, 2014), and accidents are more common in children from deprived neighbourhoods (Public Health England, 2014). In those who have an accident, head injuries are common and the majority of fatalities involve a head injury (Royal Society for the Prevention of Accidents, 2014), thus signalling the importance of helmet use at this age. In the current study, reported use of cycle helmets was low and use of reflective clothing very low amongst children whether or not they had attended Lifeskills. Only 37% of helmet owners reported wearing a helmet on their most recent cycle ride, and less than 4% of cycle owners had worn reflective or fluorescent clothing on their most recent ride. Cycle helmet ownership, and helmet use amongst those who owned a helmet, were more common in children from higher SEP families, in general concordance with previous research (Williams et al., 1997, Lang, 2007). It could be argued that it is difficult for Lifeskills to have a direct effect on these kinds of safety behaviours. For example, a child who did not own a helmet or reflective clothing prior to their Lifeskills visit would need not only to learn the benefits of such items and retain that knowledge, but also to articulate that knowledge to a parent in order to persuade them to acquire the items. Limited financial resources to buy safety equipment are obstacles to a child successfully adopting safety behaviours taught to them.

Health behaviour models have long indicated that knowledge, whilst necessary, may not be sufficient to result in behaviour change as it competes with inertia and other barriers, such as a belief that a given risk is too low for concern, or that the behaviour change required is at variance with social and behavioural norms (Ajzen, 2011, Noar and Zimmerman, 2005). For example, a study of teenagers in Oxford found high levels of knowledge about the benefits of cycle helmets, with 96% believing wearing a helmet reduces the risk of head injury, but also high levels of perceived barriers to their use with 71% thinking they ‘looked ridiculous’, and many saying their friends ‘discouraged them from wearing one’ (Joshi et al., 1994). Children's attitudes towards helmets, for example in terms of their benefits and how they look, are strong determinants of whether they wear one (Lang, 2007). To be successful, interventions need to address not only knowledge but social attitudes to the behaviour in question. They also need to be relevant to young people irrespective of social background to ensure social inequalities in health and safety behaviours are not inadvertently increased (White et al., 2009).

The results for substance perceptions and use are in agreement with the need for safety education to address more than knowledge. The vast majority of ALSPAC adolescents reported that alcohol, smoking and cannabis were ‘harmful’ or ‘very harmful’ to health when aged 14, yet many were soon using these substances themselves. By 15.5 years almost one in five reported frequent drinking, almost 10% were weekly smokers, and a similar proportion reported occasional cannabis use.

This is the first evaluation of Lifeskills with relatively long-term outcomes. We have been able to examine differences in road safety behaviours, and the perceptions of the health effects, and use, of substances, between those who did and did not attend Lifeskills using a large, longitudinal population-based cohort. We were able to adjust for some potential confounders and to take into account clustering at the school and neighbourhood level. Furthermore, we have examined both self-reported outcomes and outcomes from linkage to hospital records.

Our study does however have limitations. None of the ALSPAC measures were designed a priori to evaluate Lifeskills. For the cycle safety measures, use of a cycle helmet and fluorescent/reflective clothing refer only to the last time cycled. We have no information on the purpose of that cycle ride, or the time of day. The main objective of the Lifeskills set on substances was for the child to be able to distinguish between drugs that do good (i.e. medicines) and drugs that harm, between prescription and over-the-counter medicines, and between illegal and legal drugs. Our outcome measures are not a close match to this objective, although it can be assumed that the long-term goal of teaching children about drugs that harm is ultimately to reduce their use.

Lifeskills attendance is not recorded at the individual level and so there is a risk of misclassification in attendance status (e.g. if a child moved school part way through Year 6), which would attenuate results towards the null. The analysis of A&E attendance was compromised by the lack of data for the years immediately following the ALSPAC children's visit to Lifeskills, when we would assume Lifeskills would have the greatest potential for accident reduction. Available codes in the A&E data did not make it possible to identify causes directly related to a Lifeskills outcome. As the study sample was relatively affluent, and several outcomes socially patterned, the overall prevalences could be under-estimates of risky behaviours, or over-estimates of safe behaviours, compared to adolescents in the general population. As the section of the population most likely to have accidents was under-represented in our sample (Towner et al., 2005), this will have reduced our power to detect small effects. Finally, with the exception of cycle proficiency training, we have no information on other relevant training the children may have received (e.g. drug safety lessons in school); such training may have contributed to, or masked, differences in the outcomes by Lifeskills attendance status if the likelihood of receiving other training was associated with Lifeskills attendance.

## 5 Conclusions

The topics covered by Lifeskills are relevant to this age group, and there is the potential for an intervention of this kind to improve a range of safety behaviours, and prevent accidents. The ALSPAC data give an insight into the prevalence of some safety behaviours and reported health and safety attitudes across a number of domains amongst adolescents who both did and did not attend Lifeskills. Children who attended Lifeskills were more likely to report using pedestrian crossings on their way to school than children who did not attend, and Lifeskills attendance was associated with less reported smoking and cannabis use. Data indicate low use of cycle helmets and reflective/fluorescent clothing irrespective of Lifeskills attendance, thus demonstrating the potential impact safety education interventions in this area could have. The various barriers to change are the kinds of issues which, in partnership with Lifeskills, schools could focus on in their follow-up work arising out of the Lifeskills visit.

## Figures and Tables

**Table 1:** Child and parental characteristics by Lifeskills attendance status.

		Attended Lifeskills *n* = 2544^a^ % (95% CI)	Did not attend Lifeskills *n* = 1746^a^ % (95% CI)	*p*-Value (*χ*^2^ test)
Sex	Female	55.7 (53.7–57.6)	52.2 (49.8–54.5)	0.02

Maternal education	Degree	12.6 (11.4–14.0)	17.0 (15.3–18.8)	0.001
A level	25.9 (24.2–27.6)	25.7 (23.7–27.8)	
O level	38.9 (37.0–40.8)	35.3 (33.1–37.6)	
None/vocational	22.6 (21.1–24.3)	22.0 (20.1–24.0)	

Highest parental occupational social class	I or II	57.1 (55.2–59.0)	60.0 (57.6–62.2)	0.15
III non-manual	28.2 (26.5–30.0)	25.2 (23.2–27.3)	
III manual	10.3 (9.2–11.6)	10.8 (9.4–12.4)	
IV or V	4.4 (3.6–5.2)	4.0 (3.2–5.0)	

Cycle proficiency training	Yes	40.3 (38.4–42.2)	36.1 (33.9–38.4)	0.006

a As the study sample differed by outcome, the sample for this comparison was defined as those who returned the questionnaire at 13 years 10 months and who had complete confounder data [total *n* = 4290].

**Table 2:** Road safety related outcomes by Lifeskills attendance status.

Outcome	Attended Lifeskills	*n*/*N* (%)	OR (95% CI)	
			Unadjusted	Adjusted^d^
Owns cycle helmet^a^	No	744/1226 (60.7)	Ref	Ref
	Yes	1274/2067 (61.6)	0.98 (0.84–1.15)	0.97 (0.82–1.14)
Wore cycle helmet^b^	No	266/744 (35.8)	Ref	Ref
	Yes	471/1270 (37.1)	1.04 (0.85–1.27)	1.03 (0.84–1.26)
Wore reflective or fluorescent clothing^a^	No	42/1242 (3.4)	Ref	Ref
	Yes	74/2074 (3.6)	1.06 (0.72–1.55)	1.08 (0.73–1.59)
RTA in past year as pedestrian or cyclist	No	14/1107 (1.3)	Ref	Ref
	Yes	22/1834 (1.2)	0.94 (0.47–1.86)	0.97 (0.49–1.94)
Always/mostly uses pedestrian crossings on way to school^c^	No	549/1057 (51.9)	Ref	Ref
	Yes	1064/1798 (59.2)	1.35 (1.14–1.60)	1.34 (1.13–1.59)
Wore seat belt last time in car	No	1344/1402 (95.9)	Ref	Ref
	Yes	2253/2330 (96.7)	1.24 (0.86–1.79)	1.22 (0.85–1.76)
Always wears seatbelt	No	1243/1402 (88.7)	Ref	Ref
	Yes	2106/2330 (90.4)	1.22 (0.98–1.53)	1.20 (0.96–1.50)

a Restricted to those who own their own bike.

b Restricted to those who own their own bike and helmet.

c Restricted to those who crossed at least one road on foot on way to school.

d Adjusted for child's sex, age, maternal education, and highest parental occupational social class. Cycle proficiency training also included in models for cycle helmet, fluorescent clothing, and RTA outcomes.

**Table 3:** Perceived that substance use is ‘very harmful’ to health by Lifeskills attendance status.

Outcome (substance use very harmful to health)	Attended Lifeskills	*n*/*N* (%)	OR (95% CI)	
			Unadjusted	Adjusted^a^
Regular drinking – physical health	No	865/1220 (70.9)	Ref	Ref
	Yes	1428/1995 (71.6)	1.03 (0.88–1.21)	1.03 (0.88–1.20)
Regular drinking – mental health	No	672/1220 (55.1)	Ref	Ref
	Yes	1137/1995 (57.0)	1.08 (0.94–1.25)	1.07 (0.93–1.24)
Binge drinking – physical health	No	687/1220 (56.3)	Ref	Ref
	Yes	1211/1995 (60.7)	1.18 (1.02–1.37)	1.17 (1.01–1.35)
Binge drinking – mental health	No	525/1220 (43.0)	Ref	Ref
	Yes	916/1995 (45.9)	1.12 (0.97–0.29)	1.11 (0.96–1.29)
Smoking – physical health	No	898/1220 (73.6)	Ref	Ref
	Yes	1495/1995 (74.9)	1.06 (0.90–1.25)	1.07 (0.90–1.26)
Smoking – mental health	No	461/1220 (37.8)	Ref	Ref
	Yes	795/1995 (39.9)	1.09 (0.94–1.26)	1.09 (0.94–1.26)
Cannabis – physical health	No	852/1220 (69.8)	Ref	Ref
	Yes	1437/1995 (72.0)	1.09 (0.93–1.28)	1.05 (0.89–1.23)
Cannabis – mental health	No	837/1220 (68.6)	Ref	Ref
	Yes	1399/1995 (70.1)	1.06 (0.91–1.25)	1.04 (0.88–1.21)

a Adjusted for child's sex, age, maternal education, and highest parental occupational social class.

**Table 4:** Substance use by Lifeskills attendance status.

Outcome	Attended Lifeskills	*n*/*N* (%)	OR (95% CI)	
			Unadjusted	Adjusted^a^
Frequent drinking	No	174/910 (19.1)	Ref	Ref
	Yes	267/1491 (17.9)	0.92 (0.74–1.14)	0.93 (0.75–1.16)
Binge drinking	No	87/910 (9.6)	Ref	Ref
	Yes	149/1491 (10.0)	1.06 (0.80–1.41)	1.07 (0.80–1.42)
Behavioural problems due to alcohol	No	90/910 (9.9)	Ref	Ref
	Yes	122/1491 (8.2)	0.81 (0.61–1.08)	0.83 (0.62–1.10)
Recent smoking	No	160/910 (17.6)	Ref	Ref
	Yes	212/1491 (14.2)	0.79 (0.63–0.99)	0.78 (0.62–0.98)
Weekly smoking	No	94/910 (10.3)	Ref	Ref
	Yes	114/1491 (7.7)	0.73 (0.54–0.97)	0.71 (0.53–0.96)
Occasional cannabis	No	97/910 (10.7)	Ref	Ref
	Yes	116/1491 (7.8)	0.72 (0.54–0.97)	0.74 (0.55–0.99)
Problematic cannabis use	No	28/910 (3.1)	Ref	Ref
	Yes	45/1491 (3.0)	0.98 (0.61–1.58)	1.02 (0.63–1.66)

a Adjusted for child's sex, age, maternal education, and highest parental occupational social class.
