# Supplementary material for: An evaluation of the impact of ‘Lifeskills’ training on road safety, substance use and hospital attendance in adolescence
Source: Accid Anal Prev. 2016 Jan;86:108–13. doi: 10.1016/j.aap.2015.10.017 (PMC4682169; doi:10.1016/j.aap.2015.10.017)
Supplement: Supplementary file 1 [file mmc1.docx]

**Supplementary Material**

Table A.1: Summary of Lifeskills Learning Objectives

| Main topics (set) | Learning Objectives^a^ |
| --- | --- |
| Road safety  (Urban Road) | Awareness of the effectiveness of fluorescent/reflective materials.  A realistic understanding of speed and stopping distances.  Have developed some of the skills and knowledge necessary for survival in today’s traffic.  Awareness of implications of not wearing seatbelts. |
| Consumer rights  (Supermarket) | Awareness of the law regarding the sale of some products (e.g. solvents, cigarettes, lottery cards).  Awareness of labels on foods (ingredients, sell-by dates). |
| Harmful substances, drug awareness  (Garage) | Awareness of importance of safe storage of harmful household substances.  Awareness and understanding of drugs that help and drugs that can harm. |
| Home safety, fire, 999 call  (House set 1) | Aware of potential dangers in bathroom (poisoning, drowning, scalds).  Aware of potential fire hazards in bedroom (candles, cigarettes, matches, electric blanket).  Understand importance of fitting smoke alarm.  Be able to develop fire escape plan and make 999 emergency call to fire brigade. |
| Home safety, security, crime reduction, gas escape  (House set 2) | Able to identify dangers to toddler and/or older person in living room.  Able to devise strategy for action in event of unexpected caller to door.  Identify hazards in kitchen and dining room.  Able to recognise smell of gas and know what to do if there is a leak.  Able to make telephone call to utility (TRANSCO). |
| Electrical, garden, building site safety  (Garden/Electricity sub-station/Building Site) | Aware of common dangers in garden (e.g. BBQ, paddling pool).  Awareness of dangers of electrical apparatus in home and community and potential for electrocution.  Aware that building sites are dangerous and not suitable places for children. |
| First aid, 999 call  (Playground) | Aware of what action to take when finding injured or unconscious person.  Aware of the ‘recovery position’ and had an opportunity to practice putting casualty into it.  Have had opportunity to practice making a 999 call. |
| Environment, water safety, railway safety  (River/railway) | Aware of the dangers posed by water.  Appreciation of how they can help someone in distress in water whilst remaining safe themselves.  Be aware of dangers of trespass and vandalism on the railway.  Know what action to take if obstructions/debris are found on railway track. |
| Country code, farm safety, rural road safety  (Country Lane/farm) | Awareness of road safety in rural environment.  Awareness of the provisions of the Country Code.  Have an awareness of hazards on farms. |

^a^Adapted from the Guides’ Briefing Notes, November 2000

Table A.2: Comparison of included and excluded sample

|  |  | Excluded sample n=5822^a^ | Study sample  n=4290^b^ | Comparing excluded and study sample  p-value (χ^2^ test for categorical, t test for continuous variables) |
| --- | --- | --- | --- | --- |
| Age at outcome | mean (years) | 13.9 | 13.9 | 0.26 |
|  |  |  |  |  |
| Sex | Female | 45.1 (43.8-46.3) | 54.2 (52.7-55.7) | <0.0005 |
|  |  |  |  |  |
|  |  | n=4680 |  |  |
| Maternal education | Degree | 5.7 (5.0-6.4) | 14.4 (13.4-15.5) | <0.0005 |
|  | A level | 16.5 (15.5-17.6) | 25.8 (24.5-27.1) |  |
|  |  | 35.4 (34.0-36.8) | 37.4 (36.0-38.9) |  |
|  | None/Vocational | 42.4 (41.0-43.9) | 22.4 (21.2 - 23.6) |  |
|  |  |  |  |  |
|  |  | n=4031 |  |  |
| Parental occupational social class | I or II | 42.2 (40.7-43.8) | 58.3 (56.8-59.7) | <0.0005 |
|  | III non-manual | 29.7 (28.4-31.2) | 27.0 (25.7-28.3) |  |
|  | III manual | 19.1 (17.9-20.3) | 10.5 (9.7-11.5) |  |
|  | IV or V | 9.0 (8.1-9.9) | 4.2 (3.7-4.9) |  |
|  |  |  |  |  |
|  |  | n=381 |  |  |
| Cycle proficiency training | Yes | 34.6 (30.0-39.6) | 38.6 (37.1-40.0) | 0.13 |
|  |  |  |  |  |
|  |  | n=5822 |  |  |
| Lifeskills attendance | Yes | 60.3 (59.1-61.1) | 59.3 (57.8-60.8) | 0.29 |

^a^ Excluded due to not having complete outcome and/or confounder data [outcome data: returned 13yr 10mth questionnaire; confounders: sex, age, maternal education, parental occupational class, cycle proficiency training).

^b^ Study sample consists of individuals with complete outcome and confounder data.

Table A.3: Outcomes by sex

| **Outcome** | **Boys** | | | | **Girls** | | | | | **χ^2^ p value** |
| --- | --- | --- | --- | --- | --- | --- | --- | --- | --- | --- |
|  | **n/N** | | **%** | | **n/N** | | **%** | | |  |
| **Road Safety** | | | | | | | | | | |
| Owns cycle helmet^a^ | 971/1557 | | 62.4 | | 1047/1736 | | 60.3 | | | 0.23 |
| Wore cycle helmet ^b^ | 365/969 | | 37.7 | | 372/1045 | | 35.6 | | | 0.34 |
| Wore reflective or fluorescent clothing^a^ | 70/1492 | | 4.5 | | 46/1754 | | 2.6 | | | 0.004 |
| Always/mostly uses pedestrian crossings on way to school^c^ | 654/1256 | | 52.1 | | 959/1599 | | 60.0 | | | <0.00005 |
| Wore seat belt last time in car | 1603/1679 | | 95.5 | | 1994/2053 | | 97.1 | | | 0.007 |
| Always wears seatbelt | 1479/1679 | | 88.1 | | 1870/2053 | | 91.1 | | | 0.003 |
| Road traffic accident in past year as pedestrian or cyclist | 20/1273 | | 1.6 | | 16/1668 | | 1.1 | | | 0.14 |
| **Perceived substance use as ‘very harmful’ to health** | | | | | | | | | | |
| ***Alcohol*** |  | | |  | |  | |  |  | |
| Frequent drinking - physical health | 962/1383 | | | 69.6 | | 1331/1832 | | 72.7 | 0.05 | |
| Regular drinking - mental health | 771/1383 | | | 55.8 | | 1038/1832 | | 56.7 | 0.61 | |
| Binge drinking - physical health | 805/1383 | | | 58.2 | | 1093/1832 | | 59.7 | 0.41 | |
| Binge drinking - mental health | 635/1383 | | | 45.9 | | 806/1832 | | 44.0 | 0.28 | |
| ***Tobacco*** |  | | |  | |  | |  |  | |
| Smoking - physical health | 1071/1383 | | | 77.4 | | 1322/1832 | | 72.2 | 0.0007 | |
| Smoking - mental health | 591/1383 | | | 42.7 | | 665/1832 | | 36.3 | 0.0002 | |
| ***Cannabis*** |  | | |  | |  | |  |  | |
| Cannabis - physical health | 945/13836 | | | 68.3 | | 1344/1832 | | 73.4 | 0.002 | |
| Cannabis - mental health | 943/1383 | | | 68.2 | | 1293/1832 | | 70.6 | 0.14 | |
|  |  | | |  | |  | |  |  | |
| **Use of substances** | | | | | | | | | | |
| ***Alcohol*** | |  | |  | |  | |  |  | |
| Regular drinking | | 215/1052 | | 20.4 | | 226/1349 | | 16.8 | 0.02 | |
| Binge drinking | | 114/1052 | | 10.8 | | 122/1349 | | 9.0 | 0.14 | |
| Behavioural problems | | 100/1052 | | 9.5 | | 112/1349 | | 8.3 | 0.30 | |
| ***Tobacco*** | |  | |  | |  | |  |  | |
| Recent smoking | | 125/1052 | | 11.9 | | 247/1349 | | 18.3 | <0.00005 | |
| Weekly smoking | | 61/1052 | | 5.8 | | 147/1349 | | 10.9 | <0.00005 | |
| ***Cannabis*** | |  | |  | |  | |  |  | |
| Occasional cannabis | | 107/1052 | | 10.2 | | 106/1349 | | 7.9 | 0.05 | |
| Problematic cannabis use | | 46/1052 | | 4.4 | | 27/1349 | | 2.0 | 0.0008 | |
| **A&E** | |  | |  | |  | |  |  | |
| A&E attendance | | 185/694 | | 26.7 | | 190/1074 | | 17.7 | <0.0005 | |

^a^Restricted to those who own their own bike

^b^Restricted to those who own their own bike and helmet

^c^Restricted to those who crossed at least one road on way to school

Table A.4: Outcomes by maternal education

| **Outcomes** | | **Degree** | | | **A level** | | | **O Level** | | | | | **Vocational/None** | | | | **χ^2^ p value** |
| --- | --- | --- | --- | --- | --- | --- | --- | --- | --- | --- | --- | --- | --- | --- | --- | --- | --- |
|  |  | **n/N** | | **%** | **n/N** | | **%** | **n/N** | | | **%** | | **n/N** | **%** | | |  |
| **Road Safety** | | | | | | | | | | | | | | | | | |
| Owns cycle helmet^a^ | | 305/385 | | 79.2 | 576/831 | | 69.3 | | 773/1292 | | 59.8 | | 364/785 | | 46.4 | | <0.0005 |
| Wore cycle helmet ^b^ | | 156/305 | | 51.2 | 232/576 | | 40.3 | | 254/771 | | 32.9 | | 95/362 | | 26.2 | | <0.0005 |
| Wore reflective or fluorescent clothing^a^ | | 6/391 | | 1.5 | 30/833 | | 3.6 | | 47/1305 | | 3.6 | | 33/787 | | 4.2 | | 0.13 |
| Always/mostly uses pedestrian crossings on way to school^c^ | | 191/332 | | 57.5 | 418/721 | | 58.0 | | 628/1099 | | 57.1 | | 376/703 | | 53.5 | | 0.31 |
| Wore seat belt last time in car | | 416/430 | | 96.7 | 911/943 | | 96.6 | | 1402/1454 | | 96.4 | | 868/905 | | 95.9 | | 0.83 |
| Always wears seatbelt | | 390/430 | | 90.7 | 859/943 | | 91.1 | | 1307/1454 | | 89.9 | | 793/905 | | 87.6 | | 0.08 |
| Road traffic accident in past year as pedestrian or cyclist | | 7/390 | | 1.8 | 6/738 | | 0.8 | | 16/1157 | | 1.4 | | 7/656 | | 1.1 | | 0.48 |
| **Perceived substance use as ‘very harmful’ to health** | | | | | | | | | | | | | | | | | |
| ***Alcohol*** | |  | |  |  | |  | |  | |  |  | |  | | |  |
| Regular drinking - physical health | | 298/406 | | 73.4 | 576/807 | | 71.4 | | 898/1253 | | 71.7 | 521/749 | | 69.6 | | | 0.56 |
| Regular drinking - mental health | | 218/406 | | 53.7 | 445/807 | | 55.1 | | 704/1253 | | 56.2 | 442/749 | | 59.0 | | | 0.28 |
| Binge drinking - physical health | | 242/406 | | 59.6 | 484/807 | | 60.0 | | 735/1253 | | 58.7 | 437/749 | | 58.3 | | | 0.90 |
| Binge drinking - mental health | | 166/406 | | 40.9 | 372/807 | | 46.1 | | 566/1253 | | 45.2 | 337/749 | | 45.0 | | | 0.37 |
| ***Tobacco*** | |  | |  |  | |  | |  | |  |  | |  | | |  |
| Smoking - physical health | | 326/406 | | 80.3 | 610/807 | | 75.6 | | 928/1253 | | 74.1 | 529/749 | | 70.6 | | | 0.003 |
| Smoking - mental health | | 119/406 | | 29.3 | 303/807 | | 37.6 | | 510/1253 | | 40.7 | 324/749 | | 43.3 | | | <0.0005 |
| ***Cannabis*** | |  | |  |  | |  | |  | |  |  | |  | | |  |
| Cannabis - physical health | | 269/406 | | 66.3 | 560/807 | | 69.4 | | 910/1253 | | 72.6 | 550/749 | | 73.4 | | | 0.03 |
| Cannabis - mental health | | 263/406 | | 64.8 | 553/807 | | 68.5 | | 877/1253 | | 70.0 | 543/749 | | 72.5 | | | 0.05 |
| **Use of substances** | | | | | | | | | | | | | | | | | |
| ***Alcohol*** |  | |  | | |  |  |  | |  | |  | |  | |  | |
| Regular drinking | 66/354 | | 18.6 | | | 116/654 | 17.7 | 170/912 | | 18.6 | | 89/481 | | 18.5 | | 0.97 | |
| Binge drinking | 30/354 | | 8.5 | | | 54/654 | 8.3 | 98/912 | | 10.8 | | 54/481 | | 11.2 | | 0.21 | |
| Behavioural problems | 31/354 | | 8.8 | | | 45/654 | 6.9 | 86/912 | | 9.4 | | 50/481 | | 10.4 | | 0.18 | |
| ***Tobacco*** |  | |  | | |  |  |  | |  | |  | |  | |  | |
| Recent smoking | 39/354 | | 11.0 | | | 98/654 | 15.0 | 153/912 | | 16.8 | | 82/481 | | 17.1 | | 0.06 | |
| Weekly smoking | 17/354 | | 4.8 | | | 45/654 | 6.9 | 91/912 | | 10.0 | | 55/481 | | 11.4 | | 0.001 | |
| ***Cannabis*** |  | |  | | |  |  |  | |  | |  | |  | |  | |
| Occasional cannabis | 43/354 | | 12.2 | | | 54/654 | 8.3 | 71/912 | | 7.8 | | 45/481 | | 9.4 | | 0.09 | |
| Problematic cannabis use | 9/354 | | 2.5 | | | 13/654 | 2.0 | 31/912 | | 3.4 | | 20/481 | | 4.2 | | 0.16 | |
| **A&E** |  | |  | | |  |  |  | |  | |  | |  | |  | |
| A&E attendance | 42/283 | | 14.8 | | | 104/478 | 21.8 | 137/650 | | 21.1 | | 92/357 | | 25.8 | | 0.01 | |

^a^Restricted to those who own their own bike

^b^Restricted to those who own their own bike and helmet

^c^Restricted to those who crossed at least one road on way to school

Table A.5: Outcomes by highest occupational social class

| **Outcomes** | | **I and II** | | | **IIInm** | | | **IIIm** | | | | | **IV and V** | | | | **χ^2^ p value** |
| --- | --- | --- | --- | --- | --- | --- | --- | --- | --- | --- | --- | --- | --- | --- | --- | --- | --- |
|  |  | **n/N** | | **%** | **n/N** | | **%** | **n/N** | | | **%** | | **n/N** | **%** | | |  |
| **Road Safety** | | | | | | | | | | | | | | | | | |
| Owns cycle helmet^a^ | | 1268/1845 | | 68.7 | 524/939 | | 55.8 | | 180/374 | | 48.1 | | 46/135 | | 34.1 | | <0.0005 |
| Wore cycle helmet ^b^ | | 524/1265 | | 41.4 | 143/524 | | 27.3 | | 58/180 | | 32.2 | | 12/45 | | 26.7 | | <0.0005 |
| Wore reflective or fluorescent clothing^a^ | | 58/1854 | | 3.1 | 31/954 | | 3.3 | | 18/372 | | 4.8 | | 9/136 | | 6.6 | | 0.08 |
| Always/mostly uses pedestrian crossings on way to school^c^ | | 880/1551 | | 56.7 | 478/848 | | 56.4 | | 185/319 | | 58.0 | | 70/137 | | 51.1 | | 0.58 |
| Wore seat belt last time in car | | 1996/2064 | | 96.7 | 1032/1068 | | 96.6 | | 399/426 | | 93.7 | | 170/174 | | 97.7 | | 0.01 |
| Always wears seatbelt | | 1880/2064 | | 91.1 | 956/1068 | | 89.5 | | 359/426 | | 84.3 | | 154/174 | | 88.5 | | <0.0005 |
| Road traffic accident in past year as pedestrian or cyclist | | 22/1659 | | 1.3 | 11/842 | | 1.3 | | <5/310 | | <1.0 | | <5/130 | | <1.0 | | 0.58 |
| **Perceived substance use as ‘very harmful’ to health** | | | | | | | | | | | | | | | | | |
| ***Alcohol*** | |  | |  |  | |  | |  | |  |  | |  | | |  |
| Regular drinking - physical health | | 1281/1779 | | 72.0 | 651/931 | | 69.9 | | 257/351 | | 73.2 | 104/154 | | 67.5 | | | 0.39 |
| Regular drinking - mental health | | 985/1779 | | 55.4 | 529/931 | | 56.8 | | 209/351 | | 59.5 | 86/154 | | 55.8 | | | 0.52 |
| Binge drinking - physical health | | 1058/1779 | | 59.5 | 550/931 | | 59.1 | | 207/351 | | 59.0 | 83/154 | | 53.9 | | | 0.61 |
| Binge drinking - mental health | | 786/1779 | | 44.2 | 411/931 | | 44.2 | | 175/351 | | 49.9 | 69/154 | | 44.8 | | | 0.26 |
| ***Tobacco*** | |  | |  |  | |  | |  | |  |  | |  | | |  |
| Smoking - physical health | | 1356/1779 | | 76.2 | 678/931 | | 72.8 | | 254/351 | | 72.4 | 105/154 | | 68.2 | | | 0.04 |
| Smoking - mental health | | 625/1779 | | 35.1 | 395/931 | | 42.4 | | 165/351 | | 47.0 | 71/154 | | 46.1 | | | <0.0005 |
| ***Cannabis*** | |  | |  |  | |  | |  | |  |  | |  | | |  |
| Cannabis - physical health | | 1237/1779 | | 69.5 | 693/931 | | 74.4 | | 248/351 | | 70.7 | 111/154 | | 72.1 | | | 0.06 |
| Cannabis - mental health | | 1220/1779 | | 68.6 | 669/931 | | 71.9 | | 239/351 | | 68.1 | 108/154 | | 70.1 | | | 0.32 |
| **Use of substances** | | | | | | | | | | | | | | | | | |
| ***Alcohol*** |  | |  | | |  |  |  | |  | |  | |  | |  | |
| Regular drinking | 258/1424 | | 18.1 | | | 133/658 | 20.2 | 37/224 | | 16.5 | | 13/95 | | 13.7 | | 0.33 | |
| Binge drinking | 139/1424 | | 9.8 | | | 69/658 | 10.5 | 21/224 | | 9.4 | | 7/95 | | 7.4 | | 0.79 | |
| Behavioural problems | 117/1424 | | 8.2 | | | 57/658 | 8.7 | 27/224 | | 12.1 | | 11/95 | | 11.6 | | 0.22 | |
| ***Tobacco*** |  | |  | | |  |  |  | |  | |  | |  | |  | |
| Recent smoking | 194/1424 | | 13.6 | | | 115/658 | 17.5 | 44/224 | | 19.6 | | 19/95 | | 20.0 | | 0.02 | |
| Weekly smoking | 101/1424 | | 7.1 | | | 64/658 | 9.7 | 32/224 | | 14.3 | | 11/95 | | 11.6 | | 0.002 | |
| ***Cannabis*** |  | |  | | |  |  |  | |  | |  | |  | |  | |
| Occasional cannabis | 130/1424 | | 9.1 | | | 50/658 | 7.6 | 25/224 | | 11.2 | | 8/95 | | 8.4 | | 0.41 | |
| Problematic cannabis use | 41/1424 | | 2.9 | | | 16/658 | 2.4 | 14/224 | | 6.3 | | <5/95 | | <3.0 | | 0.03 | |
| **A&E** |  | |  | | |  |  |  | |  | |  | |  | |  | |
| A&E attendance | 228/1079 | | 21.1 | | | 112/453 | 24.7 | 25/171 | | 14.6 | | 10/65 | | 15.4 | | 0.03 | |

^a^Restricted to those who own their own bike

^b^Restricted to those who own their own bike and helmet

^c^Restricted to those who crossed at least one road on way to school

**Figure A.1: Sample flow diagram**


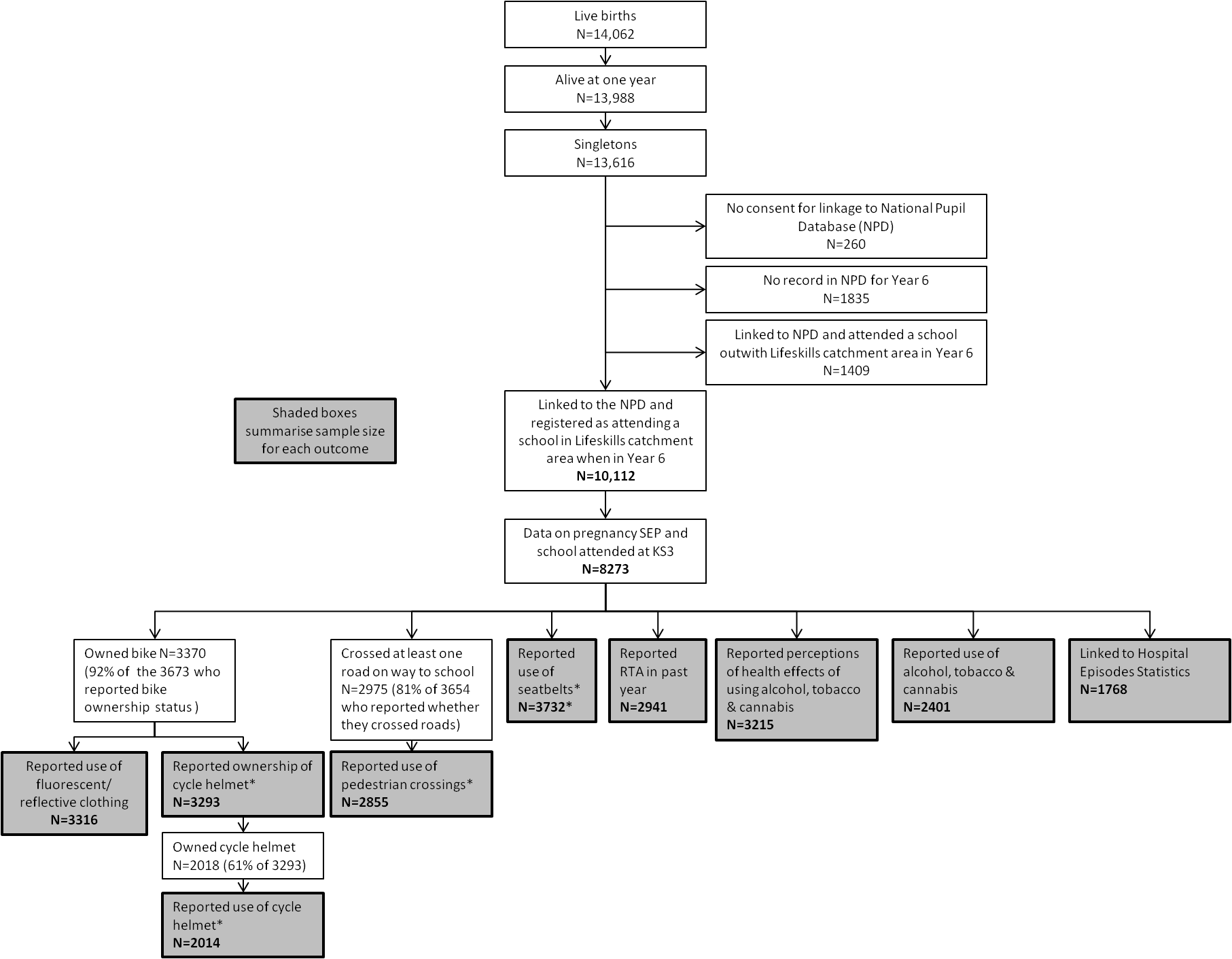


*For these outcomes, multilevel models included both school attended and neighbourhood (LSOA) of residence and so sample is further restricted to those who have data on LSOA of residence (this restriction reduced each sample by approximately 60, compared to when LSOA was not included).
